# Development of a conceptual framework to underpin a health-related quality of life outcome measure in paediatric chronic fatigue syndrome/myalgic encephalopathy (CFS/ME): prioritisation through card ranking

**DOI:** 10.1007/s11136-019-02399-z

**Published:** 2020-01-06

**Authors:** Roxanne M. Parslow, Nina Anderson, Danielle Byrne, Kirstie L. Haywood, Alison Shaw, Esther Crawley

**Affiliations:** 1 Centre for Academic Child Health (CACH), Bristol Medical School, University of Bristol, 1-5 Whiteladies Road, Bristol, BS8 1NU UK; 2 Warwick Research in Nursing, Division of Health Sciences, Warwick Medical School, University of Warwick, Coventry, CV4 7AL UK

**Keywords:** Chronic fatigue syndrome/myalgic encephalopathy (CFS/ME), Adolescents, Conceptual framework, Health-related quality of life (HRQoL), Patient-reported outcome measure (PROM), Qualitative

## Abstract

**Purpose:**

Chronic fatigue syndrome (CFS)/myalgic encephalopathy (ME) is relatively common in children and is disabling at an important time in their development. This study aimed to develop a conceptual framework of paediatric CFS/ME using the patient-perspective to ensure that the content of a new outcome measure includes the outcomes *most* important to young people.

**Methods:**

We developed a child-centred interactive card ranking exercise that included health-related quality of life (HRQoL) outcomes identified from a previous review of the literature as well as qualitative work. Adolescents and their parents selected and ranked the outcomes most important to them and discussed each outcome in further detail. Adolescents were purposively sampled from a single specialist paediatric CFS/ME service in England. Interviews were audio recorded and transcribed verbatim, and thematic framework analysis was used to develop the final conceptual framework.

**Results:**

We interviewed 43 participants in which there are 21 adolescents, 12–17 years of age with mild–moderate CFS/ME and their parents (20 mothers and 2 fathers). ‘Symptoms’, ‘tiredness’, ‘payback and crashing’ and ‘activities and hobbies’ were ranked most important to improve by both children and parents. Children ranked ‘school’ higher than parents and parents ranked ‘mood’ higher than children. A youth- specific CFS/ME conceptual framework of HRQoL was produced that included 4 outcome domains and 11 subdomains: sleep, tiredness, problems concentrating, individual symptoms, fluctuation and payback, daily and general activities, participation in school, leisure and social life, mood, anxiety and self-esteem.

**Conclusions:**

An interactive card ranking exercise worked well for adolescents aged 12–17 to elicit the most important outcomes to them and explore each domain in further detail. We developed a final conceptual framework of HRQoL that forms the basis of a new paediatric patient-reported outcome measure (PROM) in CFS/ME.

## Background

Paediatric chronic fatigue syndrome/myalgic encephalopathy (CFS/ME) is relatively common (0.6–2.4% of children) [1–6], diagnosed by extreme disabling fatigue and one or more physical and/or cognitive symptoms such as sleep problems, pain, problems concentrating, headaches, sore throat and dizziness persisting for 3 months in children [7, 8]. Children can become bedbound [9], miss school [10, 11] and develop mood disorders [12, 13]. In a large cross sectional study, adolescents with CFS/ME were found to have significantly lower health-related quality of life (HRQoL) (particularly in physical and school functioning) compared to healthy controls [10]. UK clinical guidelines [8] recommend that children are offered cognitive behavioural therapy (CBT), graded exercise therapy (GET), activity management or components of each. There are no objective tests for diagnosis or recovery in CFS/ME. Therefore, patients’ subjective perceptions and experiences of their symptoms and functioning are important outcomes. Evidence of the effectiveness of treatment is hindered by the lack of well-developed patient-reported outcome measures (PROMs) for children. A review of PROMs completed by children with CFS/ME identified 13 PROMs, six were child specific and seven were not. No CFS/ME child-specific measures were identified. Evidence of essential measurement properties such as test–retest reliability, structural validity and data quality was missing for all 13 measures. The authors failed to recommend a PROM due inadequate evidence of quality and acceptability to children with CFS/ME [14].

Patient-reported outcome measures which seek to assess HRQoL are increasingly used to measure outcomes in clinical trials [15, 16]. HRQoL focuses on the impact of a condition and its treatment on a patient’s physical, social and psychological functioning [17, 18]. The first stage in developing a PROM is to produce a conceptual framework with input from the patient population, to ensure the outcomes measured have face and content validity so that the PROM includes concepts that are important to children [19, 20]. Child-specific PROMs have traditionally been developed with input from health professionals or parents alone [21–23] rather than seeking to understand what is important to children themselves. Children have been recognised as “effective content experts” in PROM development [18]. Previous research with children with CFS/ME has produced a conceptual model of what it is like living with CFS/ME and broader contextual factors [24]. However, only outcomes that are most meaningful to patients should be included in a new HRQoL PROM [25]. Therefore, the aim of this study was to understand what is *most* important to include in the final conceptual framework specific to HRQoL to underpin a new short paediatric CFS/ME PROM.

## Methods

### Study design

This was part of a larger qualitative study exploring how ‘recovery’ should be defined in paediatric CFS/ME. We wanted to understand the *most* important outcomes to adolescents to include in a short PROM as well as gather enough *detail* for each outcome to develop questionnaire items. Interviewing adolescents with CFS/ME who are very tired and have cognitive difficulties poses a challenge for traditional qualitative methods. Card ranking is used in qualitative research as an ‘enabling technique’ to aid discussion and reasons for priorities [26]. Ranking has also been used in various studies with children to prioritise the order of items in a new amblyopia PROM [27] and determine which outcomes are ‘more important’, ‘somewhat important’ or ‘not important’ to children with neurodisability using a ‘Talking Mat’ [28].

**Fig. 1:**
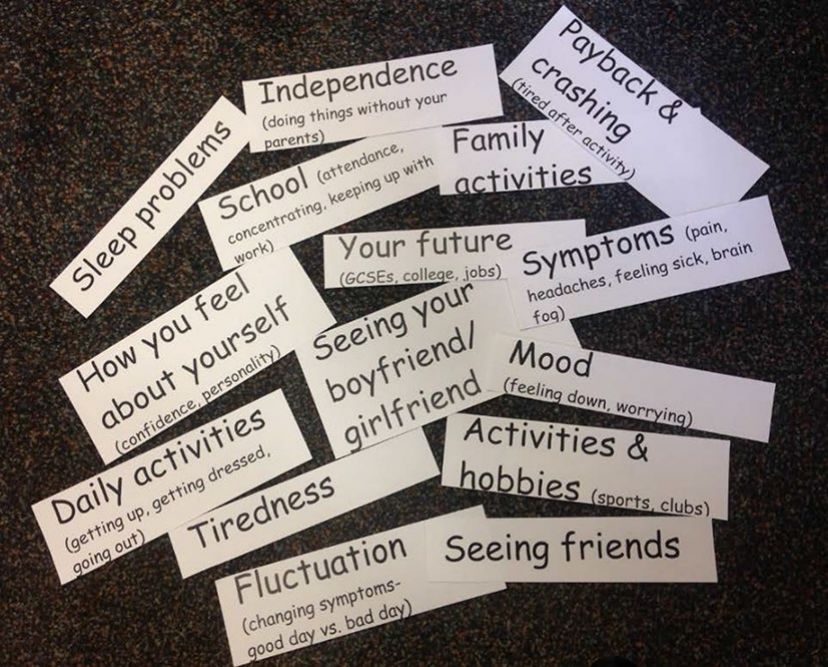
Outcome cards ranked within the interview

The semi structured topic guide was developed with a Young Person’s Advisory Group (YPAG) [29] who felt card ranking (already used in school) would allow children to prioritise the most important HRQoL outcomes [26]. Important areas of life to children affected by CFS/ME were identified from previous qualitative work [24, 30] and used to produce ‘outcome cards’ that could be ranked in order of importance within the interview (Table 1, Fig. 1). We ensured the cards included the range of HRQoL domains (physical, social and psychological). ‘Tiredness’ as the key diagnostic feature of CFS/ME was separated from other ‘symptoms’. ‘Daily activities’ was separated from ‘general hobbies’ to identify potential functional differences. We also used cards such as ‘family’, ‘friends’, ‘boyfriend and girlfriend’ and ‘independence’ that have been shown to be important to adolescents, to allow possible age differences to emerge [31]. Authors who employed a Q-sort task to prioritise 33 health outcomes for paediatric neurodisability reflected that this was challenging due to “the large number of concepts” [32]. Therefore, we were mindful to keep the list of outcomes short and broad as not to burden adolescents with CFS/ME who experience fatigue and problems concentrating.

**Table 1:** Extract from topic guide

1. Pick out the top issues/areas [cards] of your life most affected by CFS/ME	
2. Imagine you were able to improve these areas, rank these [cards] in order of what you feel is most important to improve, put the areas at the top you would most like to improve	
*Outcome cards*	
Tiredness	
Symptoms (pain, headaches, feeling sick, brain fog)	
Sleep problems	
Daily activities (getting up, getting dressed, going out)	
Payback and crashing (tired after activity)	
Fluctuation (changing symptoms—good day vs. bad day)	
School (attendance, concentrating, keeping up with work)	
Activities and hobbies (sports, clubs)	
Spending time with friends	
Family activities	
Mood (feeling down, worrying)	
How you feel about yourself (confidence, personality)	
Your future (GCSEs, college, jobs)	
Independence (doing things without your parents)	
Seeing your boyfriend/girlfriend	
PROMPTS: Why have you ranked them in that order?	
Explore dimensions of outcome cards (frequency/severity/duration/satisfaction) e.g. what symptoms bother you most, what is important about school.

Adolescents and their parents were given the cards and then asked to rank the 15 different outcomes in order of importance. The interviewer then probed further on why participants had ranked in that order and discussed each card in further detail in order to identify the dimensions of each domain (Table 1, Appendix 1).

### Participants

Qualitative studies used to develop a PROM should recruit participants that reflect as closely as possible the patient population that will use the new measure [33]. CFS/ME is more prevalent in adolescents [34], and those who are mild to moderately affected can more reliably self-report their health compared to those that are severe (housebound) where a proxy report may be required [8, 35–37]. Therefore, adolescents aged 12–17 years, diagnosed with mild to moderate CFS/ME (not housebound) [8], were recruited from a specialist paediatric chronic fatigue service in South West England. We aimed to recruit a range of participants (age, gender, and disease severity) through maximum variation purposive sampling [26].

### Data collection and analysis

Families were recruited in outpatient clinics and written consent was obtained from both parents and adolescents in person, prior to the interview. Participants were made aware that the researcher was not part of the clinical team as to avoid influencing their responses [26]. Participants were offered interviews in their own home or in the hospital and parents and adolescents were interviewed separately. Interviews continued until data saturation was reached, where new interviews produced little or no change in themes in the data [38].

Interviews were recorded on an encrypted digital audio recorder and transcribed verbatim. Thematic framework analysis [39–41] was used to develop an a priori ‘framework’ of codes based on the overall HRQoL outcome domain cards identified from previous work and used within the interview. This created an overall structure to help organise and index the data. A top-down approach was used to begin with, coding the data deductively according to the thematic framework (our knowledge of the overall outcome domains). The framework was then further developed through reading and re-reading transcripts to identify additional domains and subdomains. Inductive coding was then undertaken, with new codes derived from participants own words, or existing codes modified to provide more detail and the dimensions of the main domains. This expanded the coding framework, which served as the basis for the conceptual framework. Transcripts for the subgroups (adolescents vs parents, ages, and mild–moderate severity) were coded separately in NVIVO [42] and the groups were then compared to search for similarities and differences in the data [43, 44]. A reflective journal was kept in NVIVO to note down differences.

#### Analysis of the card ranking exercise

We identified the top five domains ranked by adolescents and parents using the card ranking. This identified the most important aspects of health impacted by CFS/ME that they wanted to improve and should be included in the conceptual framework. The qualitative data for each domain were then analysed to explore the dimensions of each domain and how to form questionnaire items.

#### Quality assurance

The first three interview transcripts were reviewed in a meeting with the three interviewers (RP, NA and DB) and a consultant CFS/ME paediatrician (EC) for interview practice (e.g. avoiding leading questions). Ten transcripts were double coded (NA) and discussed in a meeting between RP and NA to check codes were not missed as well as compare coding and interpretation in order to improve the trustworthiness of the analysis [45].

## Results

### Participants

We interviewed 43 participants: 21 adolescents and 22 parents. The age of the adolescents ranged between 12 and 17 years old, (mean 14.4 years) and the majority were female (16/21). Twenty mothers and 2 fathers participated, and one interview included both parents in a pair. Adolescents and parents were interviewed separately; however, in 4 adolescent interviews, a parent was present and mainly observed and provided support. Most interviews took place in participants own homes, one in hospital and lasted between 14 and 42 min (median 25 min).

### Outcome card ranking (Table 2)

**Fig. 2:**
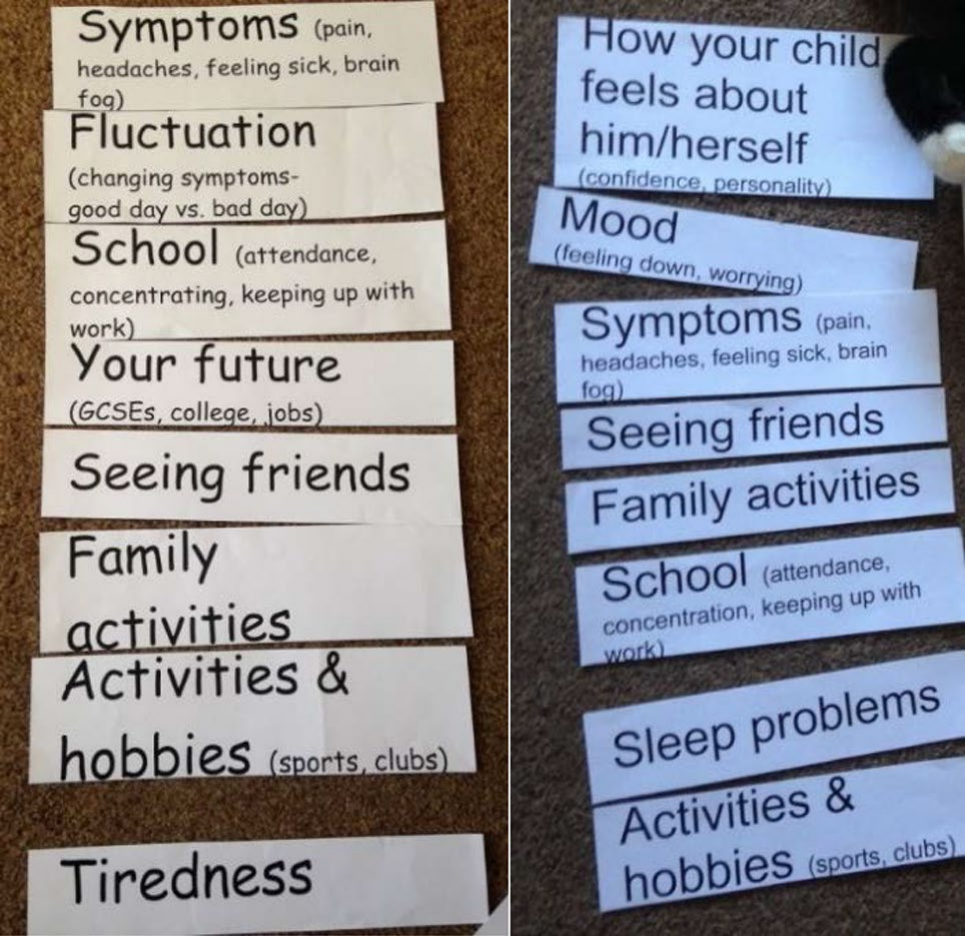
Outcome card ranking most important at top (girl 15 left, mother right)

Four outcomes were consistently ranked as important by adolescents and their parents and included in the final conceptual framework: ‘symptoms’, ‘tiredness’, ‘payback and crashing’ and ‘activities and hobbies’. ‘School’ and ‘future’ were ranked highly by adolescents. ‘Mood’ and ‘how your child feels about him/herself’ were ranked highly by parents and are also included in the final framework. Parents generally felt that their child’s health was most important to improve and that participation in school would follow (Fig. 2). Parents were more likely than their child to refer to the psychological impact of the condition. They highlighted how their child had changed from outgoing to quiet, had mood swings and experienced anxiety and depression.

**Table 2:** Top ranked outcome cards (adolescents and parents)

Adolescents			Parents		
Top ranked outcome cards	No. of pts	%	Top ranked outcome cards	No. of pts	%
Symptoms	15	71	Symptoms	13	62
School	15	71	Tiredness	12	57
Tiredness	13	62	Payback and crashing	11	52
Payback and crashing	9	43	Activities and hobbies	9	43
Your future	9	43	Mood	8	38
Activities and hobbies	7	33	How your child feels about him/herself	8	38
Friends	7	33	Sleep problems	7	33
Family	6	29	Fluctuation	7	33
Mood	6	29	School	7	33
Fluctuation	5	24	Family	5	24
Daily activities	4	19	Daily activities	3	14
Sleep problems	3	14	Friends	2	10
How you feel about yourself	3	14	Your child’s future	2	10
Independence	1	5	Independence	2	10
Boyfriend/girlfriend	0	0	Boyfriend/girlfriend	0	0

### Differences between subgroups: age and gender

Younger adolescents (12–13 years of age) ranked ‘family’ higher than older adolescents and referred to activities such as playing with toys or outside (Table 3). Older adolescents talked about leisure activities such as the cinema and 16–17-year-olds referred to wanting to be able to drive, get a job and babysit. Fourteen to 15-year-olds ranked ‘school ‘and their ‘future’ highly and referred to not being able to complete their General Certificate of Secondary Education (GCSEs) and the resulting impact of not being able to go to university or meet their full potential.

**Table 3:** Top ranked outcome cards (age groups)

Age differences								
Adolescents (12–13 years of age)			Adolescents (14–15 years of age)			Adolescents (16–17 years of age)		
Top ranked outcome cards	No. of pts	%	Top ranked outcome cards	No. of pts	%	Top ranked outcome cards	No. of pts	%
School	7	78	School	5	83	Symptoms	6	100
Payback and crashing	6	67	Symptoms	4	67	Tiredness	5	83
Tiredness	5	56	Your future	4	67	School	3	50
Symptoms	5	56	Tiredness	3	50	Payback and crashing	2	33
Family	4	44	Mood	3	50	Fluctuation	2	33
Friends	3	33	Friends	2	33	Activities and hobbies	2	33
Your future	3	33	Sleep problems	1	17	Friends	2	33
Daily activities	2	22	Daily activities	1	17	Family	2	33
Fluctuation	2	22	Payback and crashing	1	17	Your future	2	33
Activities and hobbies	2	22	Fluctuation	1	17	Sleep problems	1	17
Mood	2	22	How you feel about yourself	1	17	Daily activities	1	17
Sleep problems	1	11	Activities and hobbies	0	0	Mood	1	17
How you feel about yourself	1	11	Family	0	0	How you feel about yourself	1	17
Independence	1	11	Independence	0	0	Independence	0	0
Boyfriend/girlfriend	0	0	Boyfriend/girlfriend	0	0	Boyfriend/girlfriend	0	0

**Fig. 3:**
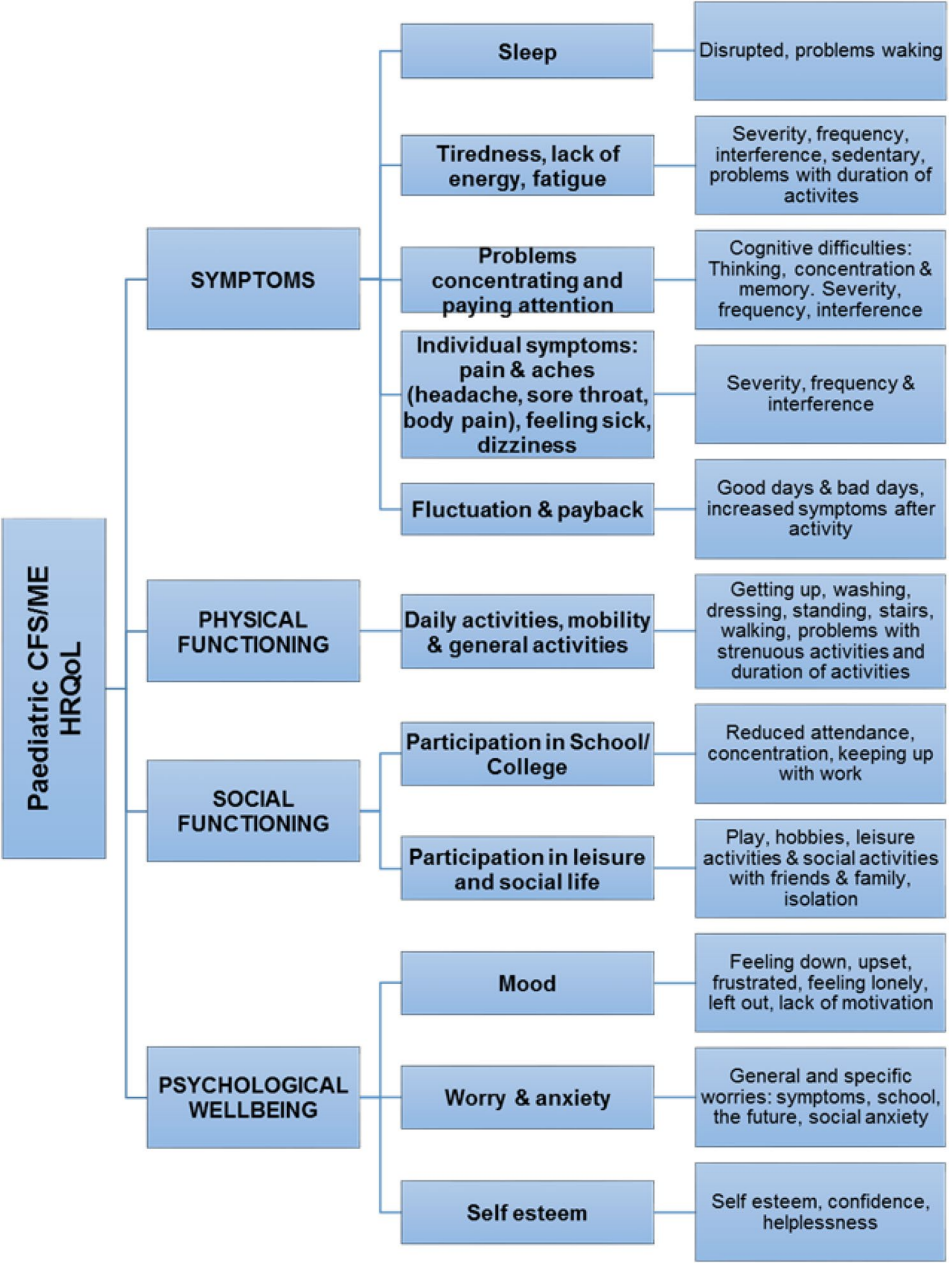
Conceptual framework of paediatric CFS/ME HRQoL

Girls ranked ‘symptoms’ higher than boys; boys ranked ‘activities and hobbies’ as more important (Table 4). Boys talked more about computer games and sports. Girls additionally valued sports but also described activities such as cooking, baking and embroidery. Boys more often referred to being ‘frustrated’ at not being able to do what they wanted to do. This range of activities is included in the dimensions as part of the final conceptual framework (Fig. 3). ‘Frustration’ is also included as a dimension of mood. Few children selected the ‘independence’ card or talked about not being able to do things without their parents. As a result, these aspects are not included in the final conceptual framework.

**Table 4:** Top ranked outcome cards by gender

Gender					
Females			Males		
Top ranked outcome cards	No. of pts	%	Top ranked outcome cards	No. of pts	%
Symptoms	14	88	School	5	100
School	10	63	Tiredness	4	80
Tiredness	9	56	Payback and crashing	2	40
Payback and crashing	7	44	Activities and hobbies	2	40
Your future	7	44	Family	2	40
Friends	6	38	Mood	2	40
Activities and hobbies	5	31	Your future	2	40
Fluctuation	4	25	Symptoms	1	20
Family	4	25	Sleep problems	1	20
Mood	4	25	Daily activities	1	20
Daily activities	3	19	Fluctuation	1	20
How you feel about yourself	3	19	Friends	1	20
Sleep problems	2	13	Independence	1	20
Independence	0	0	How you feel about yourself	0	0
Boyfriend/girlfriend	0	0	Boyfriend/girlfriend	0	0

### Conceptual framework of paediatric CFS/ME HRQoL

The final paediatric CFS/ME HRQoL conceptual framework includes 4 domains and 11 subdomains (Fig. 3). All domains and dimensions are described below with supporting quotes (Table 5) to illustrate how the final dimensions are derived from qualitative themes.

**Table 5:** Qualitative quotes from children and parents for each domain of the conceptual framework

HRQoL outcome domain	Child and parent quotes
Symptoms	
Sleep	“Until you rectify the sleep pattern then he’s not going to get better” (RP17, mother of male aged 15) “So you can go to sleep, and you don’t have a restful sleep, so you wake up and you’re like I just want to go back to bed.” (RC8, female, aged 17)
Tiredness, lack of energy, fatigue	“…it just it feels like I’m really worn out but I haven’t done anything.” (RC9) “just fall or where I just need to sit down wherever I was” (RC12, male, aged 13) “I don’t know really, I think because I’m tired I can only do a certain amount of hours” (RC6, female, aged 15)
Problems concentrating and paying attention	“…with chronic fatigue you just kind of can’t concentrate for the same amount of time” (RC10, female, aged 13). “brain fog, and you can’t like concentrate properly” (RC12, male, aged 13); “forgotten where you were” (RC12, male, aged 13);
Individual symptoms	“I think that if I like, some of the symptoms calmed down a bit then I might be able to do a bit more. Like, if I got less dizzy and stuff, and like, if I felt less sick and stuff maybe like I’d be able to go out and do a bit more” (RC3, female, aged 13) “I have back pain, which varies where it is. Like sometimes it’s on my lower back, other times on the top of my back, sometimes it’s the whole of my back, and then it will just be in the spine, and that can be really quite bad. Some days that can be really bad. It’s always there but sometimes it’s sort of cope-able, and other days it’s less cope-able, and I have my leg pain, which is the wobbly but also just sort of hurts and feels like you’ve run a marathon” (RC15, female, aged 16) : “….as bad as it can get, like, I won’t be able to eat very much, like” (RC3, female, aged 13)
Fluctuation and payback	“I would say the fluctuations from day to day are a lot less, so I have a lot more good days and a lot less bad days” (RC13, female, aged 17) “When it’s worse I get a lot more sensitive to the sound and noise, and my legs tend to get quite a lot worse, and I get a lot more dizzy” (RC15, female, aged 16) “You don’t really know when you’re going to feel okay and when you’re not,… yeah it’s just a bit frustrating” (RC18, female, aged 17) …”the next day if she does too much then she’ll get that payback, and she’ll be aching all day, her feet will hurt all day, she’ll have headaches or throat all day, and it’s just a nightmare.” (RP6, mother of female, aged 15)
Physical functioning	
Daily activities, mobility and general activities	“I can’t like move to…get myself to stand up and try and get dressed. “(RC6, female, aged 15) “at one stage we were carrying her up the stairs, she didn’t have the energy.” (RP13) “we do use a wheelchair when she’s out to try and conserve energy.” (RP3, mother of female, aged 13) “…that’s one thing you definitely miss, going out on your bike. But it’s very short bursts”(RP16, male, aged 12)
Social functioning	
Participation in school/college	“Yeah. Erm, and getting back into school, ‘cause then I can do my GCSEs and get into the universities and everything.” (RC19, female, aged 15) “Like I can’t go to school and I can’t like read very well” (RC1, female, aged 14) “Interviewer: What is it about school specifically that you’d want to improve? RC14: Being able to concentrate” (RC14, female, aged 15) “Like sometimes my homework I’m just like I’m too tired, I just… and then it piles up, so yeah that’s stressful.” (RC18, female, aged 17)
Participation in social life	A good day…I can play with erm, my dolls” (RC2, female, aged 12) “to town or to the cinema or to their house instead of them always coming here.” (RC18, female, aged 17) “when it was really bad last year she didn’t really want to play her guitar” (RP14, mother of female aged 15) “…once I stopped going to school a lot of people stopped contacting me” (RC3, female, aged 13) “no friends, no social life, and she doesn’t really have anybody. She’s got about four or five friends.” (RP6, mother of female aged 15) “Family activities, so whatever we organise always has to be around what RC11 can do really, which kind of rubs everyone else up the wrong way doesn’t it.” (RP11, mother of female aged 16)
Psychological wellbeing	
Mood	“when it’s really bad it is really, and then I get really emotional as well, which is everything like it just feels horrible.” (RC7, female, aged 17) “…things can change very quickly with different like stuff, and that kind of gets you down quite a lot as well,… sometimes you’re like you don’t know what the point is, because you’re trying and you don’t feel you’re getting anywhere (RC15, female, aged 16) “…extreme like kicking off about stuff. Because actually that’s one thing that he doesn’t really do since he’s got a bit better” (RP16, mother of male aged 12)
Worry and anxiety	“He will have anxiety so he’ll want to change his room around maybe, or it will show itself in that, or he will be not able to sleep at all.” (RP17, mother of male aged 15) “I don’t really wanna leave the house, ‘cause I think I might be ill.” (RC19, female, aged 15) “Going to college, I don’t know why I get anxious about that, and talking in class and all that side.” (RC14, female, aged 15) “RC21: And sometimes I do worry a lot, I just feel sad for no reason. Interviewer: Yeah, and worrying, is that- what is that about? RC21: Erm, my future, so what I’m going to get in GCSEs, what happens if I fail this subject or this subject?” (RC21, male, aged 15)
Self-esteem	“I just didn’t feel very good at all about myself, so no point really, because you can’t do anything, there’s no point trying or anything with anything.” (RC11, female, aged 16) “…and self-esteem. Feeling she’s not um, she feels that she’s not achieving academically, that she’s not liked, she’s got no friends, everybody hates her.” (RP10, mother of female aged 17) “She just seems sort of devoid of any energy, listless, draws into herself a little bit, goes into her shell”. (RP8, father of female aged 17)

Adolescents and parents described a range of symptoms: sleep problems, tiredness, cognitive symptoms and individual physical symptoms.

1. *Sleep* reversed sleep (sleeping in the day and not at night), problems getting to sleep, waking up in the night and trouble waking up in the morning- feeling “dead”.
2. *Tiredness* feeling “worn out”, “heavy”, “weak” and “drained”. Tiredness was often constant and could get worse at times. Only doing activities for a short time, suddenly needing to stop/sit down, being very sedentary.
3. *Problems concentrating* “Brain fog”- being unable to think, forgetting things, unable to concentrate enough in school, to read or do homework.
4. *Individual physical symptoms* pain all over the body or specific locations (head, throat, arms, legs, back). Pain, dizziness, nausea and problems eating were more frequent on worse days and very debilitating.

The presentation, impact and priorities for improvement of symptoms in adolescents were complex. Adolescents wanted to improve how severe and frequent symptoms were and the interference with their daily lives. Some adolescents specified being able to cope with mild constant symptoms but not with more severe symptoms. For some, tiredness was most important to improve whereas others wanted to get rid of headaches or nausea. All participants wanted to reduce the fluctuation of the symptoms (more good days) and the how symptoms often got worse after activity (payback).

### Physical functioning

Adolescents and their parents described how symptoms interfered with their ability ‘to do things’ and participate in daily life. They referred to a range of activities they found problematic: self-care, movement, going up and down stairs, walking, sport and managing several activities in one day. Some adolescents could only do activities for short durations and often did not leave the house. Parents accommodated adolescents to help conserve their energy, always having to plan family outings, alter or stop plans altogether. Differences between those with mild versus moderate severity were demonstrated in the qualitative data. Moderately affected adolescents reflected on problems with self-care (washing, dressing) and frequently needed help from their parents. They also described being too tired to walk and many were using a wheelchair when on outings out of the house. Some of those who were mildly affected talked about problems managing several activities in a day.

### Social functioning

Education and social life were disrupted for all adolescents in this study. Time at school had been reduced and they reported not being able to go out, stay out for long and thus missing out on hobbies and social events.

1. Participation in school/college: Reduced attendance and needing to take extra breaks. The concentration and attention required in school often led to adolescents becoming very tired, falling behind with their work.
2. Participation in leisure activities and social life: Stopped/reduced sport, hobbies or leisure activities. Younger adolescents (12–13 years of age) reported restrictions to ‘play’ with siblings, toys at home or outside in their free time. Older adolescents reported not being able to go into town, shopping or to the cinema. Adolescents often became isolated as they no longer saw friends at school, through sleepovers or parties and there was a lack of understanding from friends. Parents commented that family life revolved around the ill child’s restrictions and mood. Adolescents were unable to participate in family activities (parties, long walks, going to the cinema).

### Psychological wellbeing

All adolescents in this study described an impact on their psychological wellbeing due to symptoms limiting their usual activities, with friends and family, ranging from: low mood, frustration, feeling anxious and lacking confidence.

1. *Mood* feeling “down”, “miserable” and “upset”. Parents described “mood swings”, “tantrum” and “agitated”. Being unable to do things contributed to frustration (more often expressed by males). Adolescents often conveyed a lack of enjoyment of activities and fluctuation and payback led to feelings of hopelessness.
2. *Worry and anxiety* general anxiety and specific worries relating to: fluctuating symptoms- not knowing when symptoms will be worse, activity causing payback and being unable to cope with symptoms in social situations. Stress and anxiety in relation to GCSE’s and being unable to fulfil their potential. Adolescents often worried about the future (more evidence in 14–17 year olds) being left behind, going to university, getting a job or being able to live independently.
3. *Self-esteem, withdrawal*: staying at home, not going out with friends and wanting to be alone. Lack of confidence in carrying out activities they did previously.

## Discussion

This study includes two novel aspects: the use of a child-centred interactive card ranking technique and the resulting conceptual framework comprising the *most* important aspects of HRQoL for adolescents with CFS/ME. This outlines four outcome domains and 11 subdomains: sleep, tiredness, problems concentrating, individual symptoms, fluctuation and payback, daily and general activities, participation in school, leisure and social life, mood, anxiety and self-esteem. This conceptual framework forms the foundation of a patient-centred PROM for paediatric CFS/ME.

### Strengths and weaknesses

This was a large qualitative sample (n = 43) with adolescents recruited across the age range: 9 (12–13-year-olds), 6 (14–15-year-olds) and 6 (16–17-year-olds) to ensure the conceptual framework is representative of the range of experience. Adolescents were mostly interviewed alone (17/21) consistent with international guidance [18] and may have reduced socially desirable answers [38, 46, 47]. Parents provided an important complementary source of information incorporating their perspective in the conceptual framework [48–51]. However, patients were recruited from only one service and fewer males and moderately affected patients were recruited, which reduced opportunities to explore differences they might experience [26]. We did not sample for all patient characteristics such as socioeconomic status (SES) and ethnicity; therefore, further research is needed to identify if there are any additional HRQoL issues for different SES and ethnic minority groups.

This study employed an interactive child friendly technique following the increasing trend to use more innovative methods to understand patient experiences such as drawing, body mapping and ranking/rating [27, 28, 32, 52, 53]. The card ranking exercise, developed with feedback from adolescents, facilitated an understanding of adolescent’s priorities and preferences, and those of their parents, whilst providing a framework for deeper discussion within an interview [26]. Adolescents were in control as they ranked outcomes and this may have reduced interviewer effects [54]. The pre-defined outcome cards may have limited the domains expressed spontaneously by participants [55], however; those were carefully selected from previous extensive qualitative work and despite the option of blank cards, no new domains were added. The qualitative and quantitative data produced and the use of constant comparison helped identify differences between subgroups, such as specific problems with self-care in moderately affected adolescents [43].

### Results in context with previous literature

The domains outlined in the conceptual framework as part of this study are similar to those found in generic HRQoL models [56–58] and consistent with outcomes measured in clinical trials: pain, fatigue, physical functioning, social role participation and emotional distress [59]. This study also highlighted key aspects described by adolescents with CFS/ME: sleep problems, problems waking, fluctuating symptoms, payback (feeling tired after activity) and problems with the sustaining activities (e.g. being out all day). Parents ranked ‘mood’ highly which has been suggested as a predisposing and perpetuating factor paediatric CFS/ME [60]. This study revealed the specific types of anxieties adolescents with CFS/ME have: worries about making symptoms worse after activity or coping with symptoms in social situations. Separation anxiety and social phobia have been reported as the most elevated anxiety types in paediatric CFS/ME [13].

‘Symptoms’ were ranked as most important to improve by adolescents and parents, however, the individual experience of symptoms and which were most important to improve, varied between participants, consistent with the heterogeneity found in CFS/ME [61, 62]. Adolescents in this study also ranked ‘school’ and ‘activities and hobbies’ highly. Adolescents with CFS/ME miss an average 1 year of schooling [9] and 90% quit their hobbies [63]. This study also demonstrated the different activities important to different age groups as well as older adolescents worrying more about the future. This is consistent with the unique social and emotional aspects for different age groups [31, 64, 65]. It is advocated that adolescent outcome measures should address the importance of separation from parents [31, 66]. However, few adolescents in this study selected and rated independence from parents highly. This may be because adolescents with CFS/ME do not necessarily lose their independence completely, as they can often see friends depending on a ‘good or bad day’ or restrict the amount of time. As a result, complete ‘independence’ may be less important in this condition. This may be different for adolescents who are severely affected.

## Conclusions

The conceptual framework developed as part of this study defines the health outcomes domains that should be measured by a new paediatric HRQoL CFS/ME PROM. Adolescents describe specific impacts of symptoms (e.g. fluctuation and payback), activity (e.g. suddenly limited, problems with duration) and worries about causing payback or coping in social situations. These dimensions are not currently captured in PROMS used in paediatric CFS/ME and should be used to develop new questionnaire items [14]. When HRQoL PROMs include what matters to patients and this is reflected in healthcare, this can improve patient–clinician communication and patient adherence to treatment and promote shared decision-making for a better therapeutic alliance [67–70].

## Data Availability

The datasets used and/or analysed during the current study are available from the corresponding author on reasonable request.

## Appendix 1: extract from topic guide

### Outcome cards

*Activity: Present Young Person (YP) with outcome cards. Add any outcomes to the blank cards the YP may have spontaneously brought up. Get YP to select the areas most affected by CFS/ME and rank them in order of importance as to what is most important to improve.*

*For each card*- *explore dimensions of domains (frequency/severity/duration/satisfaction).*

*Prompts, e.g. what symptoms bother you most, what is important about school.*

*[Take a photo of order].*

1. Pick out the top issues/areas of your life most affected by CFS/ME.
2. Imagine you were able to improve these areas, rank these in order of what you feel is most important to improve, put the areas at the top you would most like to improve.
  - Tiredness
  - Symptoms (pain, headaches, feeling sick, brain fog)
  - Sleep problems
  - Daily activities (getting up, getting dressed, going out)
  - Payback and crashing (tired after activity)
  - Fluctuation (changing symptoms- good day vs. bad day)
  - School (attendance, concentrating, keeping up with work)
  - Activities and hobbies (sports, clubs)
  - Spending time with friends
  - Family activities
  - Mood (feeling down, worrying)
  - How you feel about yourself (confidence, personality)
  - Your future (GCSEs, college, jobs)
  - Independence (doing things without your parents)
  - Seeing your boyfriend/girlfriend
3. Why have you ranked them in that order?
4. How might your answer have been different a year ago? [Pick the cards].
  - Why were they important?
  - In what ways has it changed/improved?

Ending question

5. Is there anything else about having CFS/ME that you feel is important to you?

## Abbreviations

CFS: Chronic fatigue syndrome
ME: Myalgic encephalopathy
HRQoL: Health-related quality of life
PROM: Patient-reported outcome measure
YPAG: Young Person’s Advisory Group
